# Binocular advantage for prehension movements performed in visually enriched environments requiring visual search

**DOI:** 10.3389/fnhum.2014.00959

**Published:** 2014-11-28

**Authors:** Roshani Gnanaseelan, Dave A. Gonzalez, Ewa Niechwiej-Szwedo

**Affiliations:** Visuomotor Neuroscience Lab, Department of Kinesiology, University of WaterlooWaterloo, ON, Canada

**Keywords:** reaching and grasping movements, eye-hand coordination, binocular vision, visual search, phoria

## Abstract

The purpose of this study was to examine the role of binocular vision during a prehension task performed in a visually enriched environment where the target object was surrounded by distractors/obstacles. Fifteen adults reached and grasped for a cylindrical peg while eye movements and upper limb kinematics were recorded. The complexity of the visual environment was manipulated by varying the number of distractors and by varying the saliency of the target. Gaze behavior (i.e., the latency of the primary gaze shift and frequency of gaze shifts prior to reach initiation) was comparable between viewing conditions. In contrast, a binocular advantage was evident in performance accuracy. Specifically, participants picked up the wrong object twice as often during monocular viewing when the complexity of the environment increased. Reach performance was more efficient during binocular viewing, which was demonstrated by shorter reach reaction time and overall movement time. Reaching movements during the approach phase had higher peak velocity during binocular viewing. During monocular viewing reach trajectories exhibited a direction bias during the acceleration phase, which was leftward during left eye viewing and rightward during right eye viewing. This bias can be explained by the presence of esophoria in the covered eye. The grasping interval was also extended by ~20% during monocular viewing; however, the duration of the return phase after the target was picked up was comparable across viewing conditions. In conclusion, binocular vision provides important input for planning and execution of prehension movements in visually enriched environments. Binocular advantage was evident, regardless of set size or target saliency, indicating that adults plan their movements more cautiously during monocular viewing, even in relatively simple environments with a highly salient target. Nevertheless, in visually-normal adults monocular input provides sufficient information to engage in online control to correct the initial errors in movement planning.

## Introduction

Vision provides important sensory input during performance of upper limb movements, such as reaching and grasping for objects or when using tools (Jeannerod et al., 1995; Elliott et al., 2001, 2010; Goodale and Westwood, 2004). Even seemingly simple motor behaviors require several stages of information processing involving a complex interaction between the cognitive, perceptual, sensory, and motor systems. For example, the act of picking up one's favorite coffee mug located among other mugs can be characterized by at least 3 stages of information processing: (1) visual search to find the mug, (2) localization of the mug in three dimensional space in order to plan reaching and grasping, and (3) online control during movement execution. Historically, the inquiry into these components of information processing has been conducted separately (Hayhoe and Rothkoph, 2011). However, acting in the real world depends on the coordinated interaction among the perceptual, sensory, and motor systems. Thus, the main goal of our research is to examine goal-directed movements performed in visually stimulating, three-dimensional (3D) environments. Since optimal movement control requires depth perception, the specific aim of the current study was to examine the contribution of binocular vision during execution of prehension movements in a visually rich environment containing multiple objects.

Over the years, studies from different disciplines have examined the contribution of binocular vision to the performance of perceptual and motor tasks, for example, discrimination of camouflaged objects, object recognition, and upper limb prehension movements (Howard, 2012). Benefits associated with two frontally placed eyes with overlapping visual fields can arise from two separate mechanisms: binocular summation (i.e., the similarities between the images) and binocular disparity (i.e., the differences in the retinal images between the two eyes) (Howard and Rogers, 2002). It has been shown that binocular summation is an important mechanism that contributes to more efficient performance of complex motor tasks, such as bead threading and water pouring (Jones and Lee, 1981). The second mechanism that can contribute to a binocular advantage is binocular disparity, which is the basis of stereopsis and provides information about relative depth and object structure/shape. Several studies used dichoptic viewing to examine the contribution of binocular disparity to object recognition and scene processing (Edelman and Bulthoff, 1992; Bennett and Vuong, 2006; Burke, 2006). Overall, results are in agreement and show reduced errors and shorter reaction time when objects are presented stereoscopically rather than on a flat, two-dimensional (2D) surface. In addition, this advantage seems to be greater when subjects are asked to recognize objects presented from a different viewpoint. In natural environments objects are often seen from different viewpoints and might be partially occluded by other objects; thus, binocular vision might facilitate the recognition of the target object and reduce visual search time.

The first set of studies into the role of binocular vision during prehension movements were conducted by Servos and Goodale (Servos et al., 1992; Servos and Goodale, 1994). They showed that removal of binocular vision resulted in a longer latency to initiate the movement, lower peak velocity, longer movement time, especially in the deceleration phase, and smaller peak aperture. It was concluded that binocular vision provides important sensory input for both reach planning and execution. It is important to note that binocular viewing does not always provide a significant advantage during motor task performance. For example, Coull and colleagues (Coull et al., 2000) found that the kinematics of aiming movements were comparable during monocular and binocular viewing. However, binocular advantage was found in a task where the localization difficulty was increased by varying target position on a trial-by-trial basis, and the opportunity to use online or terminal feedback was also eliminated by removing the target from view upon movement initiation. Although the authors did not examine the source of the aiming errors in the monocular condition, it is possible that subjects mislocalized targets due to phoria (i.e., the deviation of the covered eye). Previous studies with visually-normal people have shown that phoria has a significant effect on direction judgments (Ono and Gonda, 1978; Ono and Weber, 1981; Park and Shebilske, 1991), thus, it would not be surprising that aiming movements executed without visual or tactile feedback exhibit phoria-related errors. On the other hand, experiments where visual feedback is provided during movement execution found no significant end-point errors (Niechwiej-Szwedo et al., 2012). Collectively, these studies indicate that the planning errors due to phoria during monocular viewing must be corrected using online feedback. To our knowledge, no previous studies have considered the temporal dynamics of this correction process. Thus, one of the aims of our study is to examine the effect of phoria on trajectory corrections during our prehension task.

Over the last 20 years, research from different laboratories has extended the initial findings and showed that binocular viewing provides a greater advantage in more complex environments, for example, when multiple objects are present (Jackson et al., 1991), when reaching unfamiliar/novel objects (Marotta and Goodale, 2001; Keefe and Watt, 2009), or when online corrections are required (Bradshaw and Elliott, 2003; Hu and Knill, 2011). Furthermore, programming of the grasping component of a prehension movement is disrupted to a greater extent in comparison to the transport phase during monocular viewing (Watt and Bradshaw, 2000; Melmoth and Grant, 2006). In short, the literature indicates that the benefits of binocular vision during planning and execution of prehension movements may be greater in visually-rich environments, and thus, it is important to investigate the significance of binocular vision using naturalistic paradigms.

Most everyday prehension movements are performed in cluttered environments; however, only few researchers have examined prehension toward targets presented among other objects (Mon-Williams et al., 2001; Biegstraaten et al., 2003; Tresilian et al., 2005; Verheij et al., 2014). When participants were asked to reach for a block of wood with an obstacle placed at various distances from the target (3, 6, 9 cm), the influence of the obstacle depended on the target-to-obstacle distance (Mon-Williams et al., 2001). Specifically, when the obstacle was placed closer to the target, participants' reaching movements had reduced velocity and smaller peak grip aperture. In contrast, obstacles located 9 cm away from the target had no effect on reach kinematics. The authors concluded that placing obstacles near the desired object affects how a person will reach for that desired object (e.g., placement of the finger between obstacle and desired target). A recent study by Verheij and colleagues demonstrated that obstacles placed underneath the movement path seem to have little effect on the kinematics compared to those that are to the side of the desired object. (Verheij et al., 2014) Therefore, obstacles change the kinematics of reaching and grasping, but the effect is dependent on the location of the obstacles.

Natural goal-directed movements are performed in a variety of environments ranging from relatively simple (i.e., a single coffee mug on a table) to complex (i.e., coffee mug placed among other objects on a table). In the second case, the observer must find the target object, while filtering out irrelevant information. This process is referred to as visual search and requires attentional resources (Eckstein, 2011; Eimer, 2014). The level of difficulty in a visual search task has been manipulated using 2D displays of various complexities. Two factors have been shown to influence the efficiency of visual search: target saliency and the number of stimuli presented in the display. Searching for a salient target defined by a unique feature is referred to as “pop-out” search, because this type of target is easily detected even in displays that contain multiple items. In contrast, searching for a target that shares features with the distractors, such as color or shape, is called “conjunction” search. This task is more difficult and the time to find the target depends on the number of items in the display.

Most natural behaviors require visual search, that is, finding and localizing the target is necessary for the subsequent planning of goal directed movements. Furthermore, eye movements are crucial for guiding upper limb manipulation actions in 3D environments. However, there are only a few studies that examined prehension movements in visually rich environments containing multiple objects, and none of these studies examined the contribution of binocular vision. Our study was conducted to examine the contribution of binocular vision during a prehension task in the context of a visual search paradigm. To manipulate the difficulty of the visual search we manipulated the set size (i.e., the target was among 2 or 5 distractors) and target salience. Specifically, subjects were asked to reach toward a target defined by single, salient feature—color (i.e., pop-out target) or toward a conjunction target, which had the same color as the distractors. To further increase the difficulty of the visual search, we also introduced a condition where the target was presented with a salient, red-colored distractor. It was hypothesized that binocular viewing would facilitate visual search and provide more reliable cues for reach planning and execution in comparison to monocular viewing. In particular, we expected that during binocular viewing participants will demonstrate: (1) more efficient search pattern characterized by fewer gaze shifts; (2) faster reach reaction time; (3) higher peak velocity and shorter movement time. We also hypothesized that the advantage associated with binocular viewing will be most evident in the larger set size and when target's salience is reduced.

## Methodology

### Participants

Fifteen healthy, right- handed adults (age: mean = 22.1 ± 4.6 years; 10 males) participated. Handedness was determined using the Edinburgh Handedness Inventory. One volunteer was excluded because he was left-handed. All participants had self-reported normal or corrected-to-normal vision and no history of visual or ocular problems. Stereoacuity was assessed using the Randot SO-002 test, and all participants had stereoacutiy of ≤50 s of arc. All volunteers who were screened for stereoacuity achieved at least 50 s of arc and no one was excluded. Eye dominance was determined using Dolman's “hole-in-card” test. The study was approved by the Research Ethics Board at the University of Waterloo and all protocols adhered to the guidelines of the Declaration of Helsinki. Informed consent was obtained from each participant.

### Apparatus

**Figure 1:**
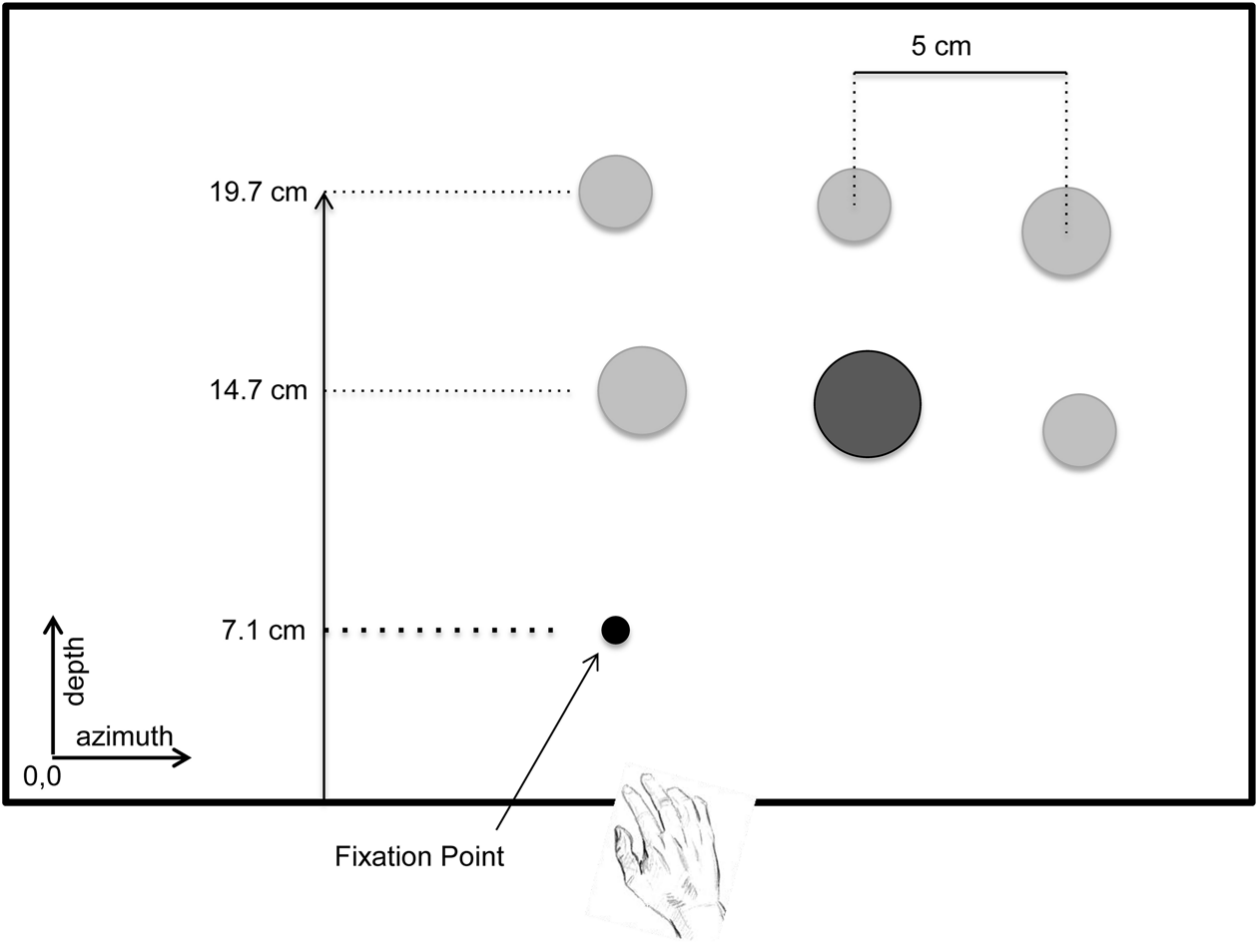
**Schematic diagram showing a bird's eye view of the workspace used in the experiment—shown here is set size 6, high salience condition**. At the beginning of each trial the hand was located at home position. The black circle represents the fixation point where the participant was required to fixate at the initiation of each trial. When the fixation point disappeared, the criterion (a circle with a diameter matching one of the pegs in the workspace) was displayed at this location. On each trial the participant was presented with three or six pegs (locations are represented by the circles in the diagram). One of the pegs matched the diameter of the criterion, and was defined as the target for that trial. Participants were instructed to reach and grasp the target as quickly as possible, and to place it on top of the criterion.

The 3D visual environment consisted of cylindrical pegs (height: 4.0 cm, diameter: 1.2, 1.6, 2.0 cm), which were arranged on a 24″ flat screen LCD monitor (Dell Professional P2312H, 1920 X 1020 @ 60 Hz). The LCD monitor was positioned horizontally and securely clamped to the table. The center of the monitor was aligned with participant's midline. The LCD display was controlled by DataPixx (VPixx Technologies, Montreal, Canada) and a VPixx script was used to randomize the placement of the pegs on the display on each trial (schematic diagram of the workspace is shown in Figure 1).

Upper limb reach kinematics were recorded with the Optotrak 3D Investigator motion capture system (Northern Digital, Waterloo, Canada) at a sampling rate of 250 Hz (spatial accuracy 0.4 mm, resolution 0.01 mm). Infrared markers were affixed to the tip of the index finger and the thumb of participant's right hand. A head-mounted EyeLink II (SR Research, Mississauga, Canada) eyetracker was used to record eye position at a sampling rate of 250 Hz (spatial accuracy 0.5°; RMS resolution 0.01°). The MotionMonitor software (Innovative Sports Technology, Chicago, USA) was used to synchronize the recording of eye and limb kinematics and to integrate the position data from the Optotrak and EyeLink into a common reference frame.

The Optotrak system was calibrated using a three-marker digitizing probe. A Cartesian coordinate system was used and defined relative to the workspace (i.e., the LCD display) used for placing the pegs. The origin was located at the left, bottom corner of the display (Figure 1). The three-dimensional system with respect to the observer was defined as follows: x-axis, horizontal plane (azimuth); y-axis, vertical plane (elevation); z-axis, median plane (depth). Calibration for the eye tracker was performed with binocular viewing using a standard 9-point grid. Validation was performed to ensure the reliability of the calibration was <1° error. Targets for eyetracker calibration were presented on a 19″ CRT monitor (Viewsonic P95f+, 1600 × 1200 @ 87 Hz) at a viewing distance of 80 cm.

### Experimental procedure

Participants were seated at a table and executed prehension movements with their right arm. Each trial began with the participant's eyes closed and their index in a standard initial position, which was aligned with their midline. The initial posture for the thumb was not standardized, that is, participants placed their thumb in a position that was comfortable. While the participant's eyes were closed, the experimenter placed the pegs on the display in a unique grid that was provided by the VPixx script. Participants were instructed to open their eyes when they heard a warning tone and then to fixate on a fixation point presented on the LCD display for 120 ms. The fixation point was located 7.1 cm in front of their initial hand position. Subsequently, the fixation point disappeared and a criterion target was shown at the same location. The criterion was a circle which varied in diameter (1.2, 1.6, 2.0 cm). The participant's task was to find the peg that corresponded to the size of the criterion, to pick up the peg and place it on top of the criterion. On each trial the target peg was embedded among distractor pegs and there was only one peg that matched the criterion's size. Each participant was instructed to complete the task as quickly as possible.

The complexity of the visual environment was manipulated in two ways. First, the set size was either small (3 pegs) or large (6 pegs). Second, the salience of the target was either high (i.e., the target peg had a different color, which was red—high-salience condition) or low (i.e., target was the same color as the distractor—low-salience condition). On a small number of trials (8/56), a salient distractor (i.e., red-colored peg which was not the target) was presented on the display (invalid condition). The salient distractor was always presented in the same row as the target and at the farthest location from the target peg along the azimuth.

There were three viewing conditions, which were randomized in blocks among participants: (1) binocular, (2) monocular with right eye, and (3) monocular with left eye. During monocular viewing, an infrared (IR) long-pass filter was placed in front of the eye. The IR filter blocked visual input to the covered eye, but allowed the eye tracker to record its position. Each viewing condition consisted of 56 trials. On each trial the pegs were arranged in a different grid with two repetitions of each grid per viewing condition.

### Data analysis

First, reaching performance was quantified by calculating the number of errors (i.e., picking up the wrong peg). The frequency of errors was compared between each viewing condition using Pearson's Chi-square statistic. The effect of set size and target salience was also examined within each viewing condition using Pearson's Chi-square statistic.

Analysis of limb and gaze kinematic data was conducted offline using a custom-written Matlab script (Matlab, MathWorks, Natick, USA). Eye and hand position data were filtered using a second-order, dual-pass Butterworth filter with a cut-off frequency of 15 Hz for the hand data and 80 Hz for the eye data. Eye and reach velocity were obtained using a 2-point differentiation method using instantaneous velocity (adjacent data points) on the cumulative distance traveled (Hansen et al., 2008). The hand velocity data were used to obtain acceleration, also using 2-point differentiation. Reach initiation was identified when the velocity of the reach vector *exceeded* 0.02 m/s for 20 Consecutive milliseconds. The end of the movement was identified when the vector velocity *fell below* 0.1 m/s for 20 consecutive milliseconds (Elliott et al., 2006; Glazebrook et al., 2009). All trials were inspected visually to ensure that movement initiation and termination were identified correctly by the software. Movement kinematics presented in this paper (i.e., peak acceleration, peak velocity, duration of acceleration and deceleration phase) were calculated on position data obtained in the primary axis of the reaching movement (i.e., the z-axis). Due to a technical difficulty with a trigger, reach reaction time could not be obtained for 5 out of the 15 participants. Therefore, the analysis of 2 outcome measures (reach reaction time and primary gaze shift latency) is based on data obtained from 10 participants.

**Figure 2:**
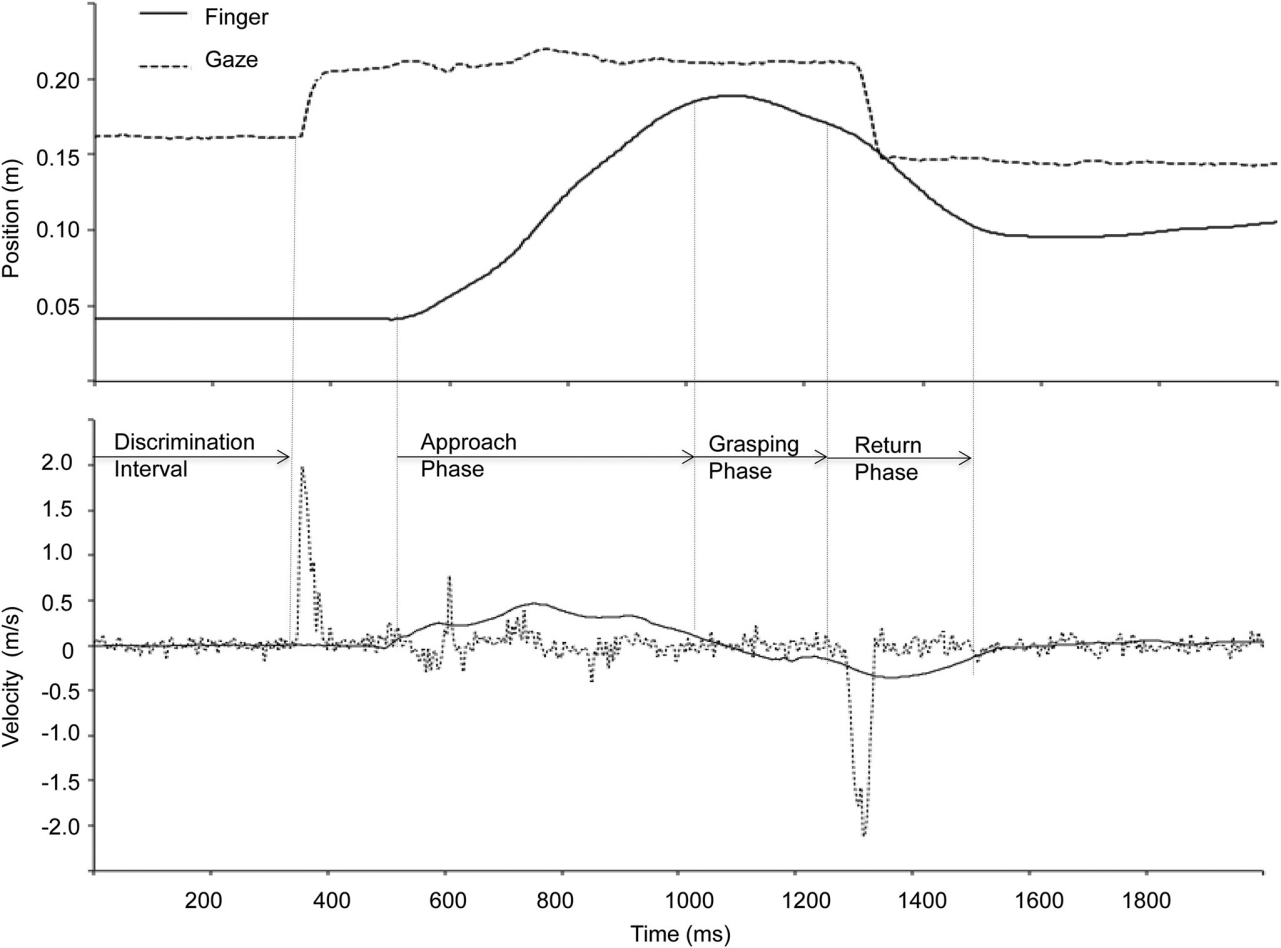
**Finger and gaze position along the depth direction (z-axis) and corresponding velocity representing a single trial during binocular viewing**. The discrimination interval is defined from the onset of the criterion to the onset of the first gaze shift. The three components of the prehension movement were identified based on the kinematic data as shown in the figure (see text for details).

The total prehension movement consisted of 3 phases: (1) the reach approach phase (i.e., transport toward the target), (2) the grasping phase, and (3) the return phase. The approach phase, defined here as the interval from reach initiation to when the velocity *fell below* 0.1 m/s for at least 20 ms along the primary direction of movement (i.e., the z-axis), the grasping phase was defined as the interval from the end of the approach phase to when the velocity *exceeded* 0.02 m/s for at least 20 ms, and the return phase was defined as the interval from the end of the grasping phase to the end of movement (Figure 2).

Gaze shifts were detected using the eye velocity data. Data were plotted and examined visually to identify saccades, which were marked manually. Saccade initiation was identified when the velocity exceeded 20°/s for a minimum duration of 20 ms. In the case of blinks, data obtained 100 ms before and after the pupil's occlusion were excluded from analysis. During binocular viewing the eye chosen for analysis was the one that provided the less noisy data. The seeing eye was used for eye movement analysis during monocular viewing.

Statistical analyses were performed using the SAS 9.2 software package. Descriptive statistics are reported as the mean and its corresponding standard deviation. All continuous measures were submitted to a repeated-measures ANOVA with 3 within-subject factors: Viewing Condition (binocular, monocular left eye, and monocular right), Set Size (set 3, set 6), and Target Salience (high, low, and invalid). Main effects and interactions were analyzed further using Tukey-Kramer *post-hoc* tests to adjust for multiple comparisons. The results section below reports the significant effects related to our hypotheses. The complete output of the analysis is presented in a Supplementary Table.

## Results

### Performance errors

Participants picked up the wrong peg 4.2% of all trials. There were significantly fewer errors [χ_(df = 2)_ = 6.6, *p* = 0.037] during binocular viewing (2.7%) in comparison to monocular viewing with the right eye (4.5%) or the left eye (5.3%). Table 1 shows the distribution of errors across experimental conditions. These data clearly show that participants were more likely to pick up the wrong peg during monocular viewing when the target was embedded in a larger set size and when the target was more difficult to discriminate (low salience and invalid condition).

**Table 1:** **Frequency (%) and total number (*n*) of errors (picking up the wrong peg)**.

	**Binocular (*n* = 21)**			**Monocular right eye (*n* = 36)**			**Monocular left eye (*n* = 43)**		
	**High salience**	**Low salience**	**Invalid**	**High salience**	**Low salience**	**Invalid**	**High salience**	**Low salience**	**Invalid**
Set 3	1.8 (3)	1.8 (3)	1.8 (1)	0.6 (1)	1.8 (3)	6.9 (4)	2.2 (4)	3.9 (7)	3.4 (2)
Set 6	1.9 (3)	4.2 (7)	7.4 (4)	6.4 (11)	5.2 (9)	^*^14.0 (8)	2.8 (5)	^*^9.5 (16)	^*^15.8(9)

* *p < 0.05 (obtained from Chi-square analysis within each viewing condition)*.

Since errors picking up the wrong peg were made on a relatively small percentage of trials, the analysis presented in the subsequent section is based on the kinematic data obtained in the correct trials.

### Gaze shifts during the acquisition phase

The participant's task was to discriminate the size of the criterion stimulus presented after fixation and to execute a reaching movement to pick up the peg whose diameter matched it. We defined the acquisition phase from the onset of the criterion to the initiation of the reaching movement. Information processing during this phase was quantified by examining the latency of the primary gaze shift and the number of scanning eye movements executed prior to reach initiation. The latency of the primary gaze shift is indicative of the time that it takes to discriminate the size of the criterion, which will be referred to as the discrimination interval. There was no significant difference between viewing conditions for the duration of the discrimination interval. The latency of primary gaze shift was 328 ± 70 ms during binocular viewing, 350 ± 73 ms during right eye viewing, and 345 ± 73 ms during left eye viewing. The fixation duration before the initiation of the secondary gaze shift was not significantly different among viewing conditions (binocular: 170 ± 73 ms; right eye: 198 ± 99 ms; left eye: 185 ± 87 ms. Primary gaze shift latency was significantly longer in the larger set size [351 ± 78 ms vs. 332 ± 73 ms; *F*_(1, 9)_ = 7.87, *p* = 0.021; η^2^ = 0.64]. Fixation duration before initiation a secondary shift was also longer in the larger set size [192 ± 92 ms vs. 172 ± 100 ms; *F*_(1, 14)_ = 18.31, *p* < 0.001; η^2^ = 0.72].

A secondary gaze shift prior to reach initiation was present on 22% of all trials. Chi-square analysis showed no significant difference between viewing conditions for the frequency of secondary gaze shifts [χ_(df = 2)_ = 0.02, *ns*]. The effect of set size and target salience was also examined within each viewing condition. Table 2 shows the frequency of secondary gaze shifts across experimental conditions. Across all viewing conditions, the frequency of a secondary gaze shifts increased when the target could not be easily discriminated (i.e., low salience and invalid condition), which was evident for displays with a larger set size in all viewing conditions, as well as during right eye monocular viewing in set size 3, invalid condition.

**Table 2:** **Frequency (%) of trials with a secondary gaze shift prior to reach initiation across viewing conditions**.

	**Binocular**			**Monocular right eye**			**Monocular left eye**		
	**High salience**	**Low salience**	**Invalid**	**High Salience**	**Low salience**	**Invalid**	**High salience**	**Low salience**	**Invalid**
Set 3	8.8	8.2	7.1	5.9	8.3	15.0^*^	6.9	9.9	12.5
Set 6	10.9	18.4^*^	15.5^*^	11.5	16.7^*^	17.5^*^	12.0	16.4^*^	17.5^*^

* *p < 0.05*.

Tertiary gaze shifts were executed on 3.9% of all trials. The frequency of these gaze shifts was similar across viewing conditions [χ_(df = 2)_ = 1.92, *ns*].

Temporal eye-hand coordination during the acquisition phase was examined by calculating the interval between the first gaze shift and reach initiation, which represents the time that was available for reach planning after the eyes were in the vicinity of the target. Analysis showed no significant difference between viewing conditions for the saccade-to-reach initiation interval. Regardless of viewing condition, participants spent a longer time planning the reaching movement after the initial gaze shift when they were presented with a larger set size [*F*_(1, 14)_ = 93.90, *p* < 0.0001; η^2^ = 0.93] and when the target couldn't be easily discriminated [*F*_(2, 28)_ = 3.80, *p* = 0.037; η^2^ = 0.21].

### Reach and grasp planning and execution

#### Temporal performance measures

**Figure 3:**
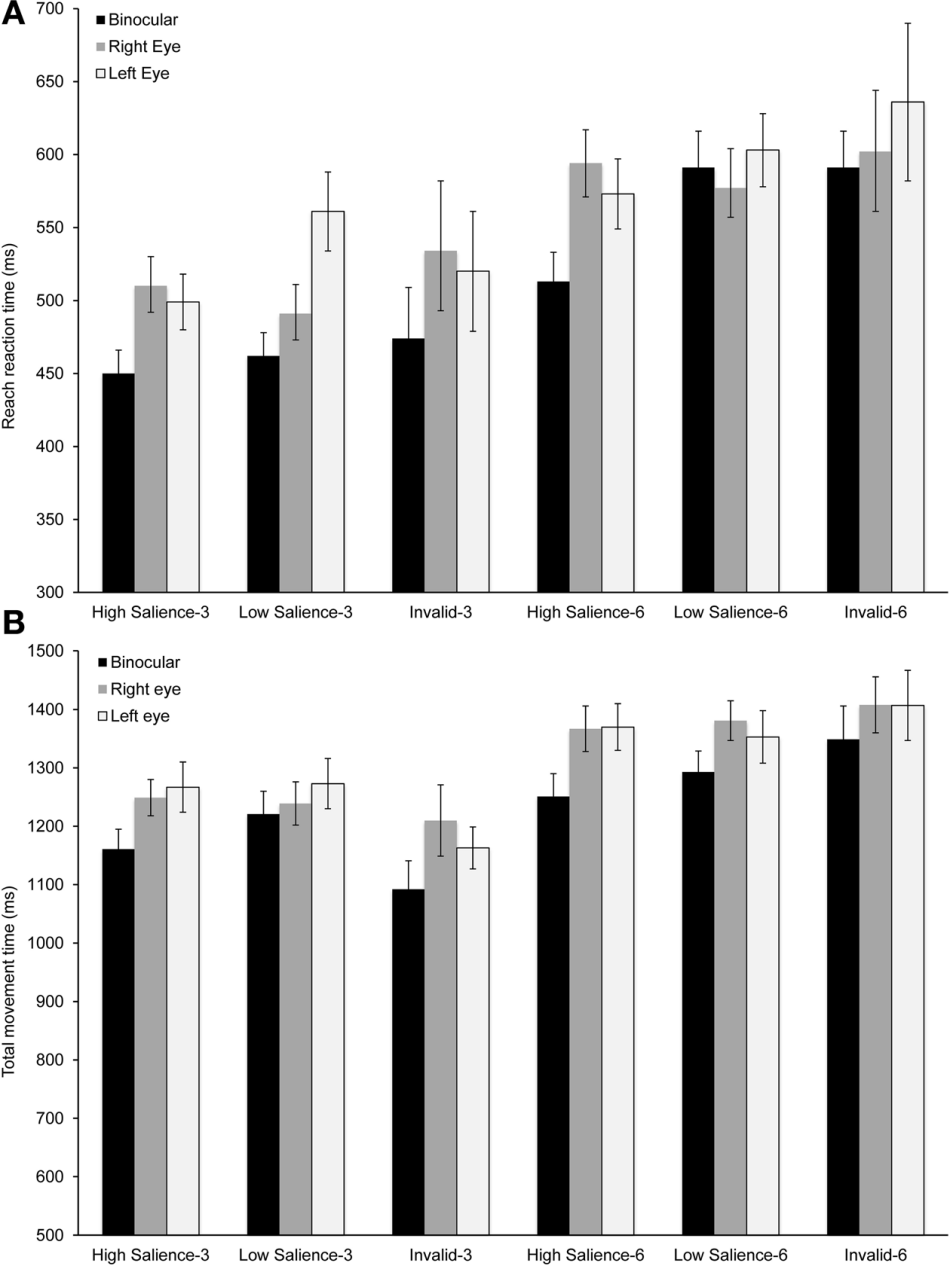
**Temporal performance measures for reaching and grasping**. **(A)** Mean reach reaction time across the experimental conditions. There was a significant main effect of viewing condition, set size, and target salience (*p* < 0.05). **(B)** Mean total movement time across the experimental conditions. There was a significant main effect of viewing condition and set size (*p* < 0.05). Error bars show ±1 standard error of the mean.

As illustrated in Figure 3A, mean reach reaction time was influenced by viewing condition, set size and target salience. The shortest response times were found during binocular viewing for most experimental conditions [*F*_(2, 18)_ = 3.58, *p* = 0.049; η^2^ = 0.28]. Increasing the difficulty of the search task by increasing the number of distractors or by reducing target salience had a similar effect across viewing conditions. Specifically, reaction time was longer for set size 6 in comparison to set size 3 [*F*_(1, 9)_ = 88.00, *p* < 0.0001; η^2^ = 0.95]. Similarly, reducing target salience resulted in longer reach reaction times [*F*_(2, 18)_ = 4.05, *p* = 0.035; η^2^ = 0.31].

The total movement time, which included the hand transport toward the target during the approach phase, the grasping phase and the return phase, was significantly shorter during binocular viewing [*F*_(2, 28)_ = 7.88, *p* = 0.002; η^2^ = 0.36; Figure 3B] and for the smaller set size [*F*_(2, 28)_ = 94.62, *p* < 0.0001; η^2^ = 0.93]. However, the effect of cue or the interactions between viewing condition and set size or viewing condition and cue were not significant. Thus, the analysis of overall performance measures supports a significant binocular advantage for movement initiation and execution. The analysis presented next focused on determining the extent of this advantage during the approach phase, the grasping phase, and the return phase.

#### Approach phase

Movement duration during the approach phase was not significantly different between the viewing conditions. However, movement time was significantly affected by set size [*F*_(1, 14)_ = 48.24, *p* < 0.0001; η^2^ = 0.87] and target saliency [*F*_(2, 28)_ = 12.65, *p* = 0.0001; η^2^ = 0.47]. The interaction between set size and target salience was also significant [*F*_(2, 28)_ = 11.99, *p* = 0.0002; η^2^ = 0.46]. *Post-hoc* tests revealed that movement times were significantly longer for the larger set size but only when a salient distractor was present.

**Figure 4:**
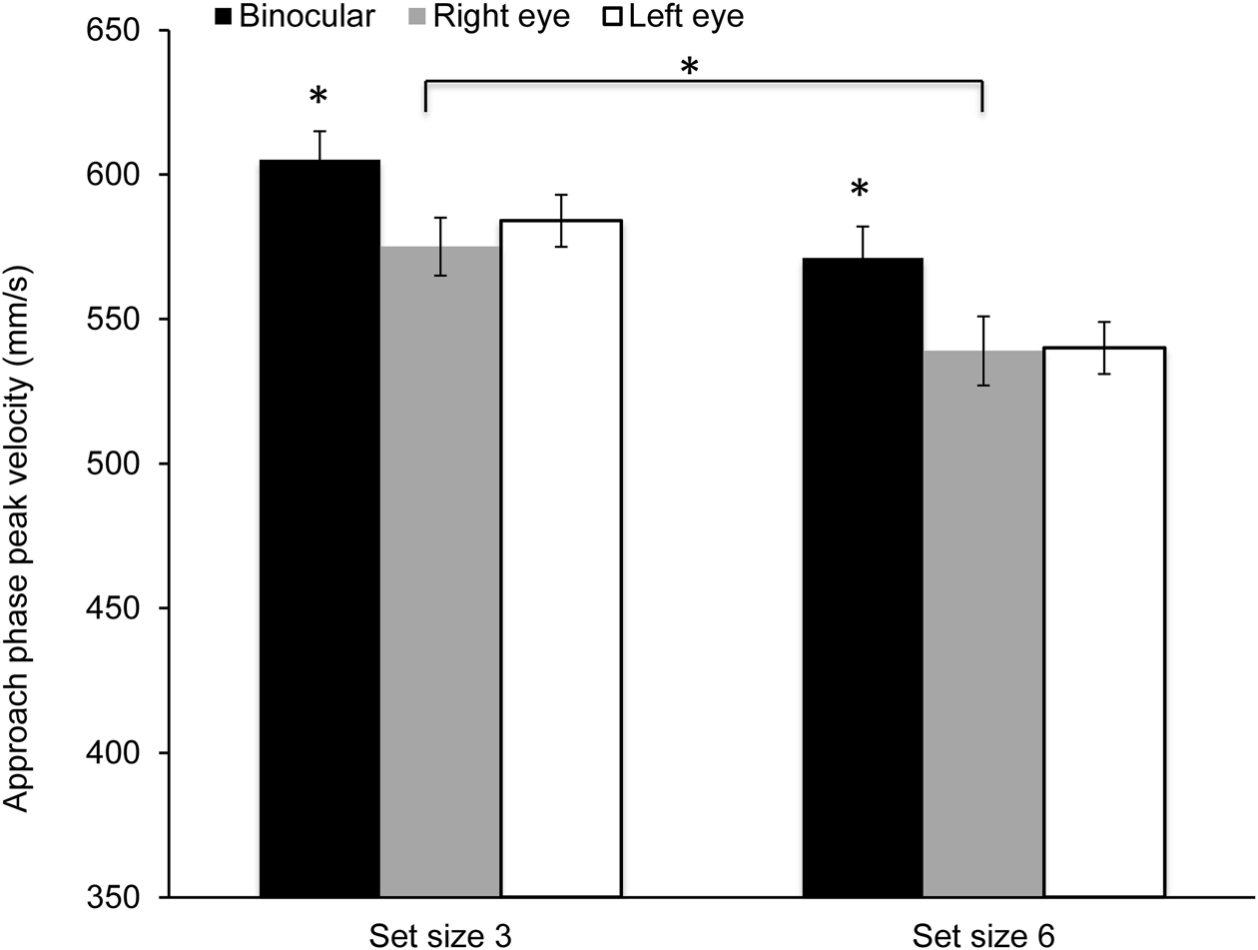
**Mean peak velocity during the approach phase along the main direction of movement (z-axis)**. Peak velocity was significantly higher during binocular viewing and in the small set size condition (^*^p < 0.05).

Figure 4 shows mean peak velocity during the approach phase. Peak velocity was significantly higher during binocular viewing [*F*_(2, 28)_ = 4.16, *p* = 0.026; η^2^ = 0.23] in comparison to monocular viewing with either eye. Peak velocities were also higher in the smaller set size [*F*_(1, 14)_ = 38.03, *p* < 0.0001; η^2^ = 0.84]. However, the main effect of cue or the interaction between viewing condition and set size or cue did not reach significance.

There were no significant differences between viewing conditions for the other reach kinematic measures during the approach phase: peak acceleration, duration of acceleration interval, peak deceleration or duration of deceleration interval. Peak acceleration and the duration of acceleration interval were influenced by set size. Specifically, peak acceleration was higher [*F*_(1, 14)_ = 19.00, *p* = 0.0007; η^2^ = 0.73] and the acceleration interval shorter [*F*_(1, 14)_ = 41.36, *p* < 0.0001; η^2^ = 0.85] in the small set size condition. The acceleration interval was also shorter in the high salience target condition [*F*_(2, 28)_ = 11.94, *p* = 0.0002; η^2^ = 0.46].

**Figure 5:**
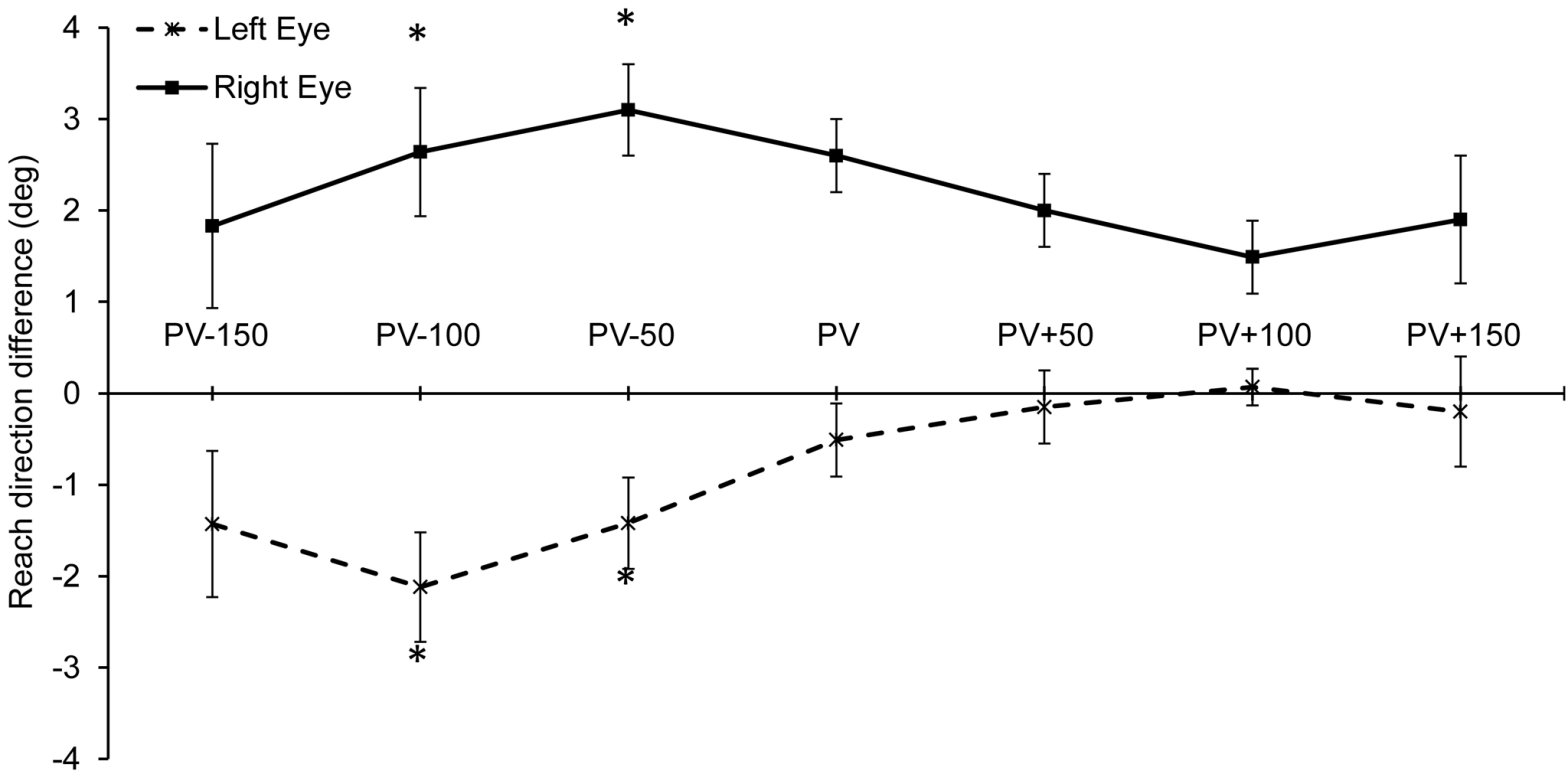
**Mean reach angle difference between monocular and binocular viewing during the approach trajectory**. A significant bias in the reach trajectory was found during the acceleration phase: 100 ms and 50 ms before reach peak velocity (i.e., at PV-100 and PV-50). (^*^p < 0.05).

Reach trajectory was examined by calculating the reach direction at peak velocity, during the acceleration phase (150, 100, and 50 ms before peak velocity) and during the deceleration phase (50, 100, and 150 ms after peak velocity). Reach direction was obtained using the finger position data along the azimuth and depth direction (reach direction = atan (x-position/z-position). In order to determine if monocular viewing introduces a bias in the reaching trajectory, we analyzed the data by subtracting the mean reach direction to each target location during binocular viewing from both monocular viewing conditions. The difference in reach direction between binocular and monocular viewing was then analyzed. Results showed that reach direction was significantly influenced by viewing condition during the acceleration phase at 100 ms before peak velocity [*F*_(1, 14)_ = 4.64, *p* = 0.049; η^2^ = 0.40], and 50 ms before peak velocity [*F*_(1, 14)_ = 7.98, *p* = 0.014; η^2^ = 0.53]. In contrast, reach direction was not reliably different between viewing conditions at peak velocity and during the deceleration phase. As shown in Figure 5, during the acceleration phase reaching trajectory had a leftward bias during left eye viewing and a rightward bias during right eye viewing.

#### Grasping phase

Grip aperture, defined as the separation between the finger and thumb, was examined at the initiation of the grasping phase. There was a significant effect of target size [*F*_(2, 28)_ = 84.61, *p* < 0.0001; η^2^ = 0.86] and set size [*F*_(2, 28)_ = 28.19, *p* = 0.0001; η^2^ = 0.80]; however, there was no significant difference between viewing conditions, and the interaction between viewing condition and target size, set size, or cue was also not significant. Regardless of viewing condition, the mean grip aperture was smaller in set size 6 in comparison to set size 3 (11.9 ± 6.5 vs.19.5 ± 9.2 mm).

**Figure 6:**
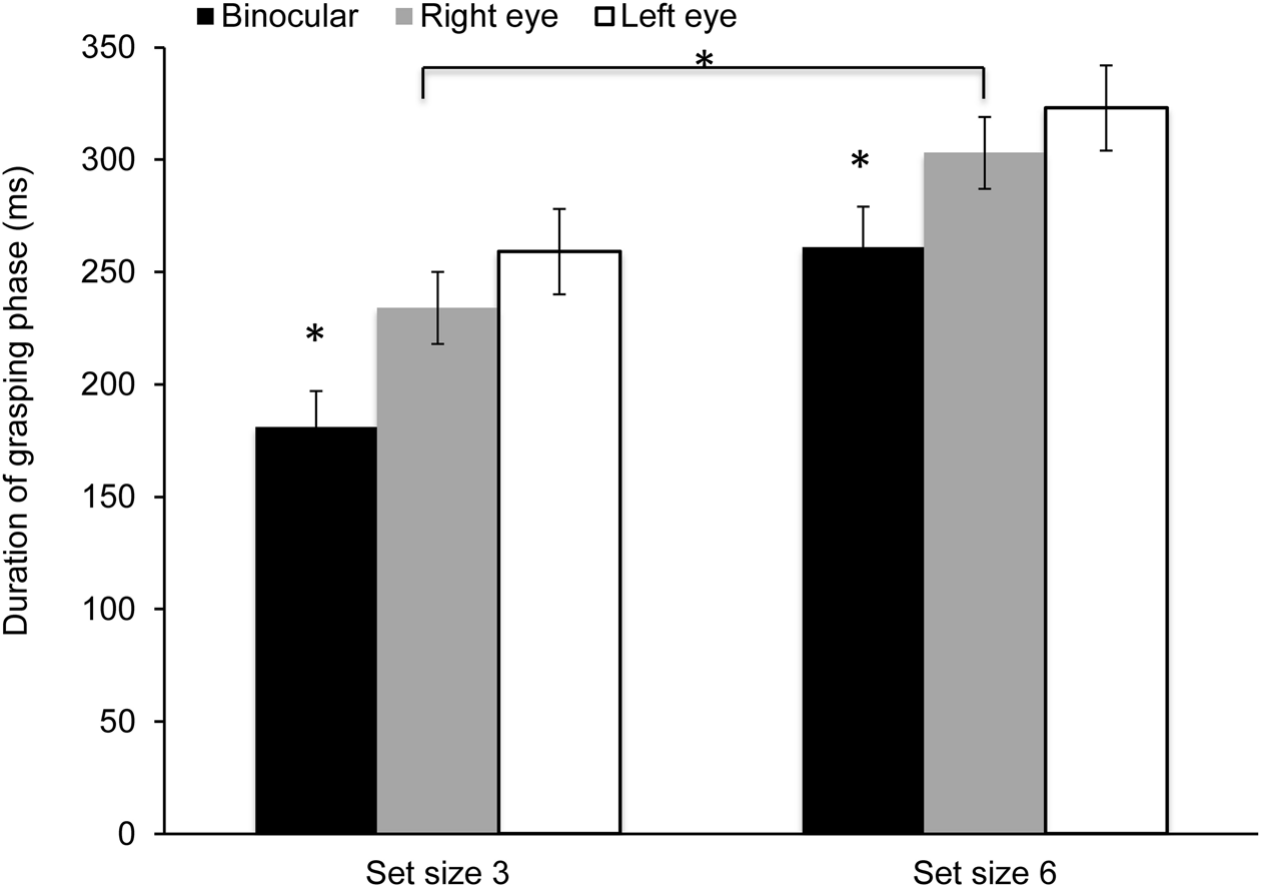
**Mean duration of the grasping phase**. The grasping interval was significantly shorter during binocular viewing and in the small set size condition (^*^p < 0.05).

Data showed a significant effect of viewing condition for the duration of time spent in the grasping phase [*F*_(2, 28)_ = 13.55, *p* < 0.0001; η^2^ = 0.49]. The effect of set size was also significant [*F*_(1, 14)_ = 128.93, *p* < 0.0001; η^2^ = 0.95]; however, the main effect of cue and the interaction did not reach significance. As shown in Figure 6, the grasping phase was prolonged during monocular viewing and in the larger set size condition.

Eye-hand coordination was assessed during grasping by calculating the interval between the end of the reaching movement and when gaze shifted away from the target. This interval represents the duration of time that participants spend fixating on the target as they were executing the grasping movement. There was a significant effect of viewing condition [*F*_(2, 28)_ = 12.63, *p* < 0.0001; η^2^ = 0.47]. Participants spent a shorter time fixating on the target prior to shifting their gaze during binocular viewing (72 ± 177 ms) in comparison to monocular viewing (right eye 197 ± 232 ms; left eye 173 ± 194 ms). No other effects were significant.

#### Return phase

There was no significant effect of viewing condition for the duration or reach kinematic during the return phase.

## Discussion

To our knowledge, this is the first study that has examined the *role of binocular vision* during prehension movements in the context of a visual search paradigm. We chose this paradigm as most reaching movements performed in natural environments are not performed toward isolated objects. Rather, one of the major requirements associated with execution of goal-directed movements is the selection of relevant objects. Moreover, the demands associated with selection of behaviorally relevant sensory information can vary substantially depending on the number of objects in the environment and the similarity among these objects. Once the object is selected, successful reaching depends on the ability to plan and execute the movement while avoiding collisions with potential obstacles. Since viewing with both eyes has been associated with performance benefits during various perceptual and motor tasks, we hypothesized that binocular vision would provide significant performance advantages during the selection process, for movement planning and execution. The main findings from this study support our hypothesis and show that reach performance is more efficient during binocular viewing, which was demonstrated by fewer errors, shorter reach reaction time, higher peak velocity and shorter grasping interval. In contrast to our hypothesis, our data showed that the advantage associated with binocular viewing did not increase in the more complex environments.

Advantages associated with binocular viewing can arise at different stages of information processing and most likely have a cumulative effect contributing to overall improvement in the performance of fine motor skills. We used a complex experimental paradigm where participants had to match the size of a three-dimensional target placed among distractors to a criterion, and then plan and execute a prehension movement. Our results showed no significant difference between viewing conditions for the latency of the primary gaze shift, which indicates that the time taken to process the visual information related to the criterion was not affected by viewing condition. This was not surprising because the criterion was a 2D shape presented at the same location as the initial fixation. However, participants made twice as many errors in picking up the wrong peg when viewing monocularly suggesting that the sensory information obtained from a 2D image was less reliable for finding a matching 3D shape, especially when the target had low salience and was embedded in a larger set size. These results are consistent with previous studies which found that subjects were significantly more accurate when asked to recognize objects presented stereoscopically in comparison to objects presented on two-dimensional displays (Edelman and Bulthoff, 1992; Bennett and Vuong, 2006; Burke, 2006). Furthermore, these studies showed that the differences between viewing conditions were greater when objects were rotated and presented from different viewpoints.

Previous studies that examined visual search using complex two-dimensional displays found that target salience and set size influence search time (Henderson et al., 2009; Beck et al., 2010; Neider and Zelinksky, 2011). That is, search times increase significantly with increasing set size and when the target shares feature(s) with the surrounding objects. We used a well-known manipulation (pop-out vs. conjunction targets) to increase the demands placed on visual processing during the selection process. Our data are consistent with the literature on visual search. We found that the frequency of gaze shifts and the time to reach initiation were both influenced by set size and target salience; however, there was no significant difference between viewing conditions. These results are in agreement with a recent study by Pomplun and colleagues (Pomplun et al., 2013). They examined visual search in a virtual 3D environment and showed that binocular disparity did not influence the search strategy. On the other hand, previous research has shown that binocular vision improves performance on tasks that require detection/discrimination of camouflaged objects (Jones and Lee, 1981; Schneider and Morglia, 1994; McKee et al., 1997). Thus, binocular advantage during search tasks is most likely dependent on the complexity of the display and would be expected in environments where objects are more difficult to discriminate. We used a visually enriched environment with multiple objects that varied in saliency, but our environment may not have been complex enough to show a binocular advantage during visual search.

Although we found no significant differences between viewing conditions in gaze behavior during the search process, the fact that reach reaction times were longer during monocular viewing indicates that binocular viewing facilitates the acquisition of sensory information for planning prehension movements in visually stimulating environments. Binocular summation is one factor that may explain faster reaction time when viewing with both eyes. Studies that used electroencephalography reported shorter latency of visual evoked potentials during binocular viewing as compared to monocular viewing (Woodman et al., 1990). Reduced latency at the first stage of information processing most likely extends to further stages of processing which include target identification and movement planning. Binocular viewing in 3D environments also activates disparity sensitive neurons in the primary visual cortex which project preferentially to parietal regions involved in reach planning and execution (Sakata et al., 1997; Fattori et al., 2004; Hajidimitrakis et al., 2011). Thus, binocular viewing might activate a more direct cortical path for planning reaching and grasping movements. Similar conclusions were also reached by a recent study which found a significant priming effect during binocular, but not during monocular viewing (Makris et al., 2013). The authors used an elegant paradigm where subjects were trained to respond with either a precision or power grip depending on the frequency of an auditory stimulus. During the test subjects were shown a real object and they were asked to respond by indicating the appropriate grip (i.e., precision or power), which was either congruent or incongruent with the priming auditory stimulus. Responses were significantly faster (20–35 ms) for congruent trials during binocular viewing, whereas a priming effect was not elicited during monocular viewing.

Most previous studies that consider the role of binocular vision during reaching and grasping have examined upper limb kinematics toward targets presented in isolation (Servos et al., 1992; Servos and Goodale, 1994; Watt and Bradshaw, 2000; Bradshaw et al., 2004; Melmoth and Grant, 2006; Keefe and Watt, 2009). The only exception is the study by Jackson et al. which examined movement kinematics in a prehension task when the target was presented with a single flanker object (Jackson et al., 1991). These authors demonstrated that the advantage associated with binocular viewing (i.e., shorter deceleration phase and smaller grip aperture) was greater in the presence of a flanker. The current study was conducted to extend the previous literature by examining prehension movements in more complex visual environments. Reaching in environments that contain multiple objects is more difficult because it requires the ability to plan a trajectory to pick up the target while avoiding the obstacles around it. Several studies have shown that reaching movements in cluttered environments are slower, have lower peak velocities, and larger grip aperture (Jackson et al., 1995; Tresilian, 1998; Mon-Williams et al., 2001; Biegstraaten et al., 2003). Binocular vision could facilitate the planning of an optimal trajectory path and online corrections in a cluttered environment. In particular, stereopsis provides unparalleled resolution of relative depth, which provides critical information about the target's shape and orientation, as well as its spatial relation with respect to the obstacles. During binocular viewing stereopsis can be combined with ocular vergence to provide the central nervous system with more accurate and more precise absolute depth information. Studies have also shown that binocular viewing provides an advantage during reach execution by facilitating online corrections (Bradshaw and Elliott, 2003; Hu and Knill, 2011). The need for online control might be increased when reaching in the presence of multiple objects because errors can arise due to mislocalization of a target in relation to the obstacles. Thus, it was expected that binocular vision would provide a greater benefit in an environment with a larger set size. Instead, our results showed that viewing condition and set size had an independent effect on prehension kinematics. Specifically, binocular vision and smaller set size were both associated with a shorter reaction time, higher peak velocity during the approach phase, and shorter total movement time. The finding that a binocular advantage was evident in both set size conditions indicates that monocular viewing provides less reliable information for planning and execution of prehension movements, even in a relatively simple environment with a target surrounded by two obstacles.

The current study provides a novel insight on the effect of monocular viewing on reach planning. Our results show that reach trajectory was biased during monocular viewing during the approach phase. Specifically, the initial direction of the reach trajectory (i.e., during the acceleration phase) was biased toward the left during left eye viewing, and toward the right during right eye viewing. This bias is consistent with the presence of esophoria during monocular viewing. Most visually-normal people experience a phoria (i.e., eye deviation) in the occluded eye, which can vary in direction and extent (Hrynchak et al., 2010). Esophoria is present when the occluded eye deviates medially and exophoria is present when the occluded eye shifts temporally. Phoria has been shown to affect the apparent direction of visual targets during monocular viewing (Park and Shebilske, 1991), during changes in accommodative vergence (Ono and Gonda, 1978), as well as during pointing tasks performed without visual feedback (Ono and Weber, 1981). Specifically, when the occluded left eye deviates medially (i.e., esophoria), the target's perceived direction will shift to the right. On the other hand, if the occluded left eye deviates temporally (i.e., exophoria), the target's apparent direction will shift to the left. Our data showed a bias in initial reaching direction that is consistent with the presence of esophoria during monocular viewing. One caveat in our current work is that the direction and extent of the phoria were not assessed in each participant. Despite this limitation, our results are in agreement with previous studies showing that target location is not perceived veridically during monocular viewing. The mislocalization of the target has a significant effect on motor planning, which is less accurate during monocular viewing. Importantly, the bias in initial trajectory was corrected shortly around the time of peak velocity which indicates that early online control was used to amend the initial reach plan. Importantly, there was no significant difference in movement time during the approach phase between viewing conditions which indicates that monocular viewing provided sufficient information to guide these early online trajectory corrections.

Previous prehension studies have shown that viewing with one eye leads to a greater grip aperture (Watt and Bradshaw, 2000; Bradshaw et al., 2004; Melmoth and Grant, 2006; Melmoth et al., 2009). In addition, Melmoth and Grant conducted a detailed analysis of the grasping phase and reported significantly greater number of errors, including larger apertures at object contact time and more adjustments of the grip aperture during monocular viewing (Melmoth and Grant, 2006). Consistent with previous literature, we found that monocular viewing had the largest effect on the grasping component of prehension, which was extended by ~20% during monocular viewing in comparison to binocular viewing. This was also accompanied by a longer fixation on the target. It is possible that monocular viewing provides less reliable cues about the object's shape or structure, and subjects might have to rely to haptic feedback to a greater extent once they contact the object. On the other hand, object features are extracted more reliably during binocular viewing which facilitates the planning and execution of reaching and grasping. Keefe and colleagues provided an alternative explanation for increased grip aperture during monocular viewing (Keefe et al., 2011). These authors used an elegant experimental paradigm to manipulate the reliability of binocular and monocular depth cues and showed that grip apertures increased when either the binocular or the monocular cues were less reliable. Optimal performance (i.e., greatest precision of size estimates and lowest grip aperture) was found when both, binocular and monocular, depth cues were available. Results from that study support that the CNS integrates multiple cues for grasp programming; however, the authors did not examine the duration of the grasping phase or gaze behavior, thus, the question that remains outstanding is whether *visuohaptic integration* is affected differentially by the reliability of depth cues during monocular and binocular viewing.

Finally, in contrast to previous studies, our results showed no reliable differences between viewing conditions for grip aperture at the initiation of the grasping phase. Instead, regardless of viewing condition, grip aperture was smaller when there were more objects in the workspace. These data are consistent with previous studies that examined reaching and grasping when obstacles are present in a workspace. For example, Mon-Williams and colleagues examined grip aperture when subjects reached toward a target presented in isolation, and with one or two obstacles (Mon-Williams et al., 2001). Their results showed that in comparison to target only condition, grip aperture decreased by 10% when one obstacle was present, and by 20% when two obstacles were present in the workspace. Importantly, the extent of the reduction was dependent on the placement of the obstacles. Our study extends the previous literature by showing that, regardless of viewing condition, subjects adopt a cautious strategy in cluttered environments by reducing their grip aperture in order to reduce the possibility of a collision.

In conclusion, we examined prehension movements in a visually rich environment where the target was embedded among distractors and reaching the target required avoiding obstacles. We found that binocular vision provides advantages during information acquisition and for reach planning and grasp execution. Furthermore, the benefit associated with binocular viewing is consistent across environments of various complexities. Overall, this study provides an important contribution to our understanding of the role of binocular vision in movement control in complex environments. This knowledge is important for developing a comprehensive neural model of motor control, and ultimately, for establishing appropriate visuomotor training protocols for people with abnormal binocular vision.

### Conflict of interest statement

The authors declare that the research was conducted in the absence of any commercial or financial relationships that could be construed as a potential conflict of interest.
