# Supplementary material for: Binocular advantage for prehension movements performed in visually enriched environments requiring visual search
Source: Front Hum Neurosci. 2014 Nov 28;8:959. doi: 10.3389/fnhum.2014.00959 (PMC4246685; doi:10.3389/fnhum.2014.00959)
Supplement: Supplementary file 1 [file Table1.PDF]

Supplementary Table: Results from statistical analysis for the kinematic outcome measures (predictors: viewing condition, set size and target salience)

	F value	DF	p-value	partial eta^2
**Reach Reaction Time**				
Viewing condition	3.58	2,18	0.049	0.282
Set size	88.00	1,9	<0.0001	0.951
Target salience	4.05	2,18	0.037	0.310
Viewing condition*Set size	0.27	2,18	0.769	0.029
Viewing condition*Target salience	0.51	4,36	0.731	0.028
**Total movement time (approach phase, grasping phase and return phase)**				
Viewing condition	7.88	2,28	0.002	0.360
Set size	94.62	1,14	<0.0001	0.931
Target salience	2.45	2,28	0.104	0.149
Viewing condition*Set size	0.45	2,28	0.080	0.031
Viewing condition*Target salience	3.57	4,56	0.013	0.113
**Reach approach duration**				
Viewing condition	3.01	2,28	0.065	0.177
Set size	48.24	1,14	<0.0001	0.873
Target salience	12.65	2,28	0.0001	0.475
Target salience*Set size	11.99	2,28	0.0002	0.461
Viewing condition*Set size	2.37	2,28	0.112	0.145
Viewing condition*Target salience	2.02	4,56	0.104	0.067
**Approach phase - reach peak velocity**				
Viewing condition	4.16	2,28	0.026	0.229
Set size	38.03	1,14	<0.0001	0.845
Target salience	3.32	2,28	0.051	0.195
Viewing condition*Set size	0.58	2,28	0.567	0.040
Viewing condition*Target salience	1.94	4,56	0.116	0.065
**Approach phase - reach peak acceleration**				
Viewing condition	2.19	2,28	0.131	0.135
Set size	19.00	1,14	0.0007	0.731
Target salience	1.77	2,28	0.189	0.112
Viewing condition*Set size	0.14	2,28	0.866	0.010
Viewing condition*Target salience	1.01	4,56	0.410	0.035
**Approach phase - duration of reach acceleration interval**				
Viewing condition	0.88	2,28	0.411	0.059
Set size	41.36	1,14	<0.0001	0.855
Target salience	11.94	2,28	0.0002	0.460
Viewing condition*Set size	0.54	2,28	0.591	0.037
Viewing condition*Target salience	1.10	4,56	0.367	0.038

	F value	DF	p-value	partial eta^2
**Approach phase - reach peak deceleration**				
Viewing condition	1.23	2,28	0.306	0.081
Set size	0.01	1,14	0.905	0.001
Target salience	0.28	2,28	0.759	0.020
Viewing condition*Set size	0.70	2,28	0.504	0.048
Viewing condition*Target salience	0.94	4,56	0.449	0.032
**Approach phase - duration of reach deceleration interval**				
Viewing condition	0.90	2,28	0.646	0.060
Set size	2.73	1,14	0.121	0.281
Target salience	0.09	2,28	0.911	0.006
Viewing condition*Set size	1.16	2,28	0.328	0.077
Viewing condition*Target salience	1.18	4,56	0.328	0.040
**Reach trajectory direction 150 ms before peak velocity**				
Viewing condition	2.16	1,14	0.164	0.236
Set size	0.76	1,14	0.397	0.098
Target salience	0.07	2,28	0.933	0.005
Viewing condition*Set size	0.92	1,14	0.353	0.116
Viewing condition*Target salience	0.52	2,28	0.602	0.036
**Reach trajectory direction 100 ms before peak velocity**				
Viewing condition	4.64	1,14	0.049	0.399
Set size	0.21	1,14	0.656	0.029
Target salience	1.29	2,28	0.291	0.084
Viewing condition*Set size	0.06	1,14	0.805	0.008
Viewing condition*Target salience	0.15	2,28	0.862	0.011
**Reach trajectory direction 50 ms before peak velocity**				
Viewing condition	7.98	1,14	0.013	0.533
Set size	0.27	1,14	0.613	0.037
Target salience	1.02	2,28	0.372	0.068
Viewing condition*Set size	0.01	1,14	0.990	0.001
Viewing condition*Target salience	0.52	2,28	0.601	0.036
**Reach trajectory direction at peak velocity**				
Viewing condition	4.48	1,14	0.053	0.390
Set size	0.28	1,14	0.606	0.038
Target salience	2.57	2,28	0.094	0.155
Viewing condition*Set size	0.13	1,14	0.725	0.018
Viewing condition*Target salience	1.03	2,28	0.370	0.069
**Reach trajectory direction 50 ms after peak velocity**				
Viewing condition	1.96	1,14	0.184	0.219
Set size	0.35	1,14	0.561	0.048
Target salience	3.54	2,28	0.042	0.202
Viewing condition*Set size	0.20	1,14	0.663	0.028
Viewing condition*Target salience	1.17	2,28	0.325	0.077

	F value	DF	p-value	partial eta^2
**Reach trajectory direction 100 ms after peak velocity**				
Viewing condition	0.74	1,14	0.403	0.096
Set size	0.19	1,14	0.668	0.026
Target salience	3.97	2,28	0.030	0.221
Viewing condition*Set size	0.32	1,14	0.583	0.044
Viewing condition*Target salience	1.35	2,28	0.276	0.088
**Reach trajectory direction 150 ms after peak velocity**				
Viewing condition	0.03	1,14	0.876	0.004
Set size	0.13	1,14	0.722	0.018
Target salience	5.88	2,28	0.007	0.296
Viewing condition*Set size	0.34	1,14	0.567	0.046
Viewing condition*Target salience	1.70	2,28	0.202	0.108
**Grasping phase - duration**				
Viewing condition	13.55	2,28	<0.0001	0.492
Set size	128.93	1,14	<0.0001	0.949
Target salience	1.10	2,28	0.347	0.073
Viewing condition*Set size	0.24	2,28	0.789	0.017
Viewing condition*Target salience	2.02	4,56	0.104	0.067
**Grasping phase - grip aperture at grasp initiation**				
Viewing condition	1.23	2,28	0.308	0.081
Set size	28.19	1,14	0.0001	0.801
Target salience	0.60	1,14	0.557	0.079
Target size	84.61	2,28	<0.0001	0.858
Viewing condition*Set size	0.20	2,28	0.823	0.014
Viewing condition*Target salience	0.59	4,56	0.668	0.021
Viewing condition*Target size	1.93	4,56	0.118	0.064
**Return phase duration**				
Viewing condition	0.12	2,28	0.887	0.008
Set size				
Target salience				
Viewing condition*Set size				
Viewing condition*Target salience				
**Return phase peak acceleration**				
Viewing condition	0.57	2,28	0.572	0.039
**Return phase peak velocity**				
Viewing condition	2.03	2,28	0.150	0.127
**Return phase duraiton of acceleration phase**				
Viewing condition	1.75	2,28	0.192	0.111

	F value	DF	p-value	partial eta^2
**Primary gaze shift latency**				
Viewing condition	1.70	2,18	0.211	0.159
Set size	7.87	1,9	0.021	0.636
Target salience	2.36	2,18	0.123	0.208
Viewing condition*Set size	1.13	2,18	0.346	0.112
Viewing condition*Target salience	0.31	4,36	0.867	0.017
**Fixation duration prior to secondary saccade**				
Viewing condition	0.23	2,28	0.796	0.016
Set size	18.31	1,14	0.0009	0.723
Target salience	0.79	2,28	0.466	0.053
Viewing condition*Set size	0.72	2,28	0.508	0.049
Viewing condition*Target salience	1.25	4,56	0.310	0.043
**Saccade-to-reach initiation interval**				
Viewing condition	1.13	2,28	0.344	0.075
Set size	93.90	1,14	0.0001	0.931
Target salience	3.80	2,28	0.037	0.213
Viewing condition*Set size	2.07	2,28	0.145	0.129
Viewing condition*Target salience	1.07	4,56	0.835	0.037
**Fixation duration during grasping phase**				
Viewing condition	12.63	2,28	<0.0001	0.474
Set size	0.01	1,14	0.977	0.001
Target salience	2.59	2,28	0.093	0.159
Viewing condition*Set size	3.01	2,28	0.060	0.177
Viewing condition*Target salience	1.19	4,56	0.324	0.041
